# Filamin A in platelets: Bridging the (signaling) gap between the plasma membrane and the actin cytoskeleton

**DOI:** 10.3389/fmolb.2022.1060361

**Published:** 2022-12-20

**Authors:** Enoli De Silva, Felix Hong, Hervé Falet, Hugh Kim

**Affiliations:** ^1^ Centre for Blood Research, University of British Columbia, Vancouver, BC, Canada; ^2^ Department of Biochemistry and Molecular Biology, University of British Columbia, Vancouver, BC, Canada; ^3^ Versiti Blood Research Institute, Milwaukee, WI, United States; ^4^ Department of Cell Biology, Neurobiology, and Anatomy, Medical College of Wisconsin, Milwaukee, WI, United States; ^5^ Department of Oral Biological and Medical Sciences, University of British Columbia, Vancouver, BC, Canada

**Keywords:** platelets, filamin A, cytoskeleton, cell signaling, actin

## Abstract

Platelets are anucleate cells that are essential for hemostasis and wound healing. Upon activation of the cell surface receptors by their corresponding extracellular ligands, platelets undergo rapid shape change driven by the actin cytoskeleton; this shape change reaction is modulated by a diverse array of actin-binding proteins. One actin-binding protein, filamin A (FLNA), cross-links and stabilizes subcortical actin filaments thus providing stability to the cell membrane. In addition, FLNA binds the intracellular portion of multiple cell surface receptors and acts as a critical intracellular signaling scaffold that integrates signals between the platelet’s plasma membrane and the actin cytoskeleton. This mini-review summarizes how FLNA transduces critical cell signals to the platelet cytoskeleton.

## Introduction

Platelets play a central role in hemostasis and wound healing (Hou et al., 2015; Etulain, 2018), and circulate in their quiescent form as discs that become activated following exposure to damaged blood vessel walls and/or soluble agonists (Jurk and Kehrel, 2005). The ligation of platelet receptors by their corresponding agonists triggers intracellular signaling pathways that result in platelet aggregation and granule secretion. The hemostatic process culminates in the conversion of the platelet “plug” into a fibrin clot (recently reviewed in (Sang et al., 2021)). Following activation, platelets change shape from a discoid to a flattened morphology characterized by multiple spike- and sheet-like cell surface extensions (Bearer et al., 2002; Sorrentino et al., 2015; Bender and Palankar, 2021).

Platelet shape change is directly mediated by dynamic nature of the actin cytoskeleton. There are two actin pools: monomeric or globular G-actin, and polymeric or filamentous F-actin (Pollard, 2016; Romero et al., 2020). The actin cytoskeleton is dynamically assembled and disassembled in response to environmental cues; actin assembly near the plasma membrane creates cell surface protrusions that mark the shape change reaction, which is critical for platelet adhesion and aggregation at sites of vascular injury (Falet et al., 2017).

A well-documented activation pathway in platelets involves the stimulation, by thrombin, of protease-activated receptor (PARs), which activates the associated G_q_ protein and its downstream effector, phospholipase C beta (PLCβ) (Michelson, 2013). Activated PLCβ then degrades phosphatidylinositol 4,5-bisphosphate (PI_4,5_P_2_) to diacylglycerol (DAG) and inositol 1,4,5-trisphosphate (I_1,4,5_P_3_), which releases calcium (Ca^2+^) from the dense tubular system thus increasing intracellular calcium levels ([Ca^2+^]_i_). DAG and Ca^2+^ activate protein kinase C (PKC) (Job and Lagnado, 1998; DeMali et al., 2003). Assembly of G-actin into F-actin near the plasma membrane creates cell surface protrusions termed filopodia and lamellipodia; this process is largely driven by the small GTPases Rac1 and Cdc42 (Azim et al., 2000; Bodie et al., 2001; Aslan and McCarty, 2013). In an alternative signaling pathway, ligation of the glycoprotein VI (GPVI) receptor by collagen also culminates in actin reorganization *via* increases in [Ca^2+^]_i_ and activation of PKC. Irrespective of the agonist, the shape change that occurs in activated platelets requires the transmission of elaborate signals from the plasma membrane to the actin cytoskeleton; this process is modulated by multiple actin-binding proteins.

BOX 1 **Key points: Actin assembly in activated platelets**

• Platelets are activated by extracellular ligands that bind to cell surface receptors.
• Activated platelets change shape during aggregation, adhesion and hemostasis.
• This shape change reaction requires efficient signal transduction between the plasma membrane and the actin cytoskeleton – mediated by actin-binding proteins.

## Filamin A (FLNA)

Actin-binding proteins can be classified as monomer-sequestering, filament-severing, bundling, and cross-linking proteins (Bearer et al., 2002). Filamins are large actin crosslinking proteins that assemble actin filaments into orthogonal networks (Stossel et al., 2001) and exist as three paralogs (A, B, and C). Filamin A (FLNA), whose gene is located on the X chromosome, is the most abundantly expressed isoform (Stossel et al., 2001), including in platelets, which also express low levels of filamin B (FLNB) whose gene resides on chromosome 3 (Takafuta et al., 1998). Filamin C (FLNC) is encoded on chromosome 7 and is primarily expressed in skeletal and cardiac muscle cells (Maestrini et al., 1993; van der Flier et al., 2002).

While a primary function of FLNA is to crosslink actin, FLNA also binds >50 other proteins including cell surface receptors, and therefore serves as a signaling scaffold (Stossel et al., 2001; Zhou et al., 2010; Zhou et al., 2021). The known FLNA binding partners, including those specifically implicated in platelet function, are listed in Table 1.

**TABLE 1:** Filamin A (FLNA) interacting partners.

Partner	Interacting domain	Relevance to platelet function	References
PKC*	FLNA Ig repeats 1–8	FLNA mediates PKC activation and promotes β1 integrin activation and cell spreading	Kim and McCulloch, (2011)
Syk	FLNA Ig repeat 5	FLNA regulates ITAM and ITAM-like-mediated signaling	Falet et al. (2010); Falet, (2013)
F-actin	FLNA Ig repeats 9–15 and Ig repeat 24	FLNA stabilizes subcortical actin filaments; crosslinking F-actin into orthogonal networks	Nakamura et al. (2007)
GPIbα	FLNA Ig repeat 17 with GPIbα cytoplasmic tail	FLNA modulates the interaction of GPIb-IX-V with vWF	Meyer et al. (1997); Williamson et al. (2002); Nakamura et al. (2006)
PKA	FLNA Ig repeat 20	PKA phosphorylates FLNA on S2152 to protect FLNA from calpain proteolysis	Chen and Stracher, (1989); Tirupula et al. (2015)
PACSIN2	FLNA Ig repeat 20 and PACSIN2-BAR domain	FLNA helps localization of PACSIN2 and regulates platelet membrane tubulation	Begonja et al. (2015)
Trio (GEF)*	FLNA Ig repeats 21–24 with GEFD1 domain of Trio	FLNA-Trio interaction induces actin-based ruffling and remodeling of cytoskeletal actin	Bellanger et al. (2000)
β3 integrin	FLNA Ig repeat 21 with β3 cytoplasmic tail	FLNA inhibits the activation of integrin αIIbβ3 by competitively blocking talin and kindlin-3 binding sites	Ma et al. (2007); Liu et al. (2015); Berrou et al. (2017)
Migfilin	FLNA Ig repeat 21	Migfilin releases the FLNA inhibition on integrin αIIbβ3 thus promoting activation	Lad et al. (2008); Zhou et al. (2020)
Pak1*	FLNA Ig repeat 23	Pak1 phosphorylates FLNA for cytoskeletal reorganization and regulates platelet activation downstream of GPVI signaling	Vadlamudi et al. (2002); Aslan et al. (2013)
FilGAP*	FLNA Ig repeat 23	FLNA-FilGAP interaction decreases Rac activity and protects cells against force-induced apoptosis	Shifrin et al. (2009); Ehrlicher et al. (2011)
STIM1	FLNA Ig repeat 24	FLNA inhibits STIM1 clustering thereby downregulating store-operated calcium entry (SOCE)	Lopez et al. (2018)
RalA*	FLNA Ig repeat 24	FLNA-RalA interaction elicits actin-rich filopodia on cell surface; RalA regulates translocation of P-selectin	Ohta et al. (1999); Wersall et al. (2018)
ROCK*	FLNA Ig repeat 24 with carboxy-terminal pleckstrin homology domain of Rock	FLNA interacts with ROCK to control actin remodeling	Ueda et al. (2003); Ohta et al. (2006); Kim and McCulloch, (2011)
RhoA*	FLNA Ig repeat 24	FLNA induces RhoA-mediated actomyosin contraction, modulates proplatelet formation, and controls megakaryocyte localization and migration	Ohta et al. (1999); Savoy and Ghosh, (2013); Sun et al. (2013); Dutting et al. (2017)
Rac*	FLNA Ig repeat 24	FLNA regulates Rac activation to control actin remodeling	Ohta et al. (1999)
Cdc42*	FLNA Ig repeat 24	FLNA-Cdc42 interaction regulates the actin cytoskeleton, and controls MK localization and migration	Ohta et al. (1999); Dutting et al. 2(017)

*Proteins whose interactions with FLNA, have been shown in other (non-platelet) cell types.

### Structure of FLNA

**FIGURE 1:**
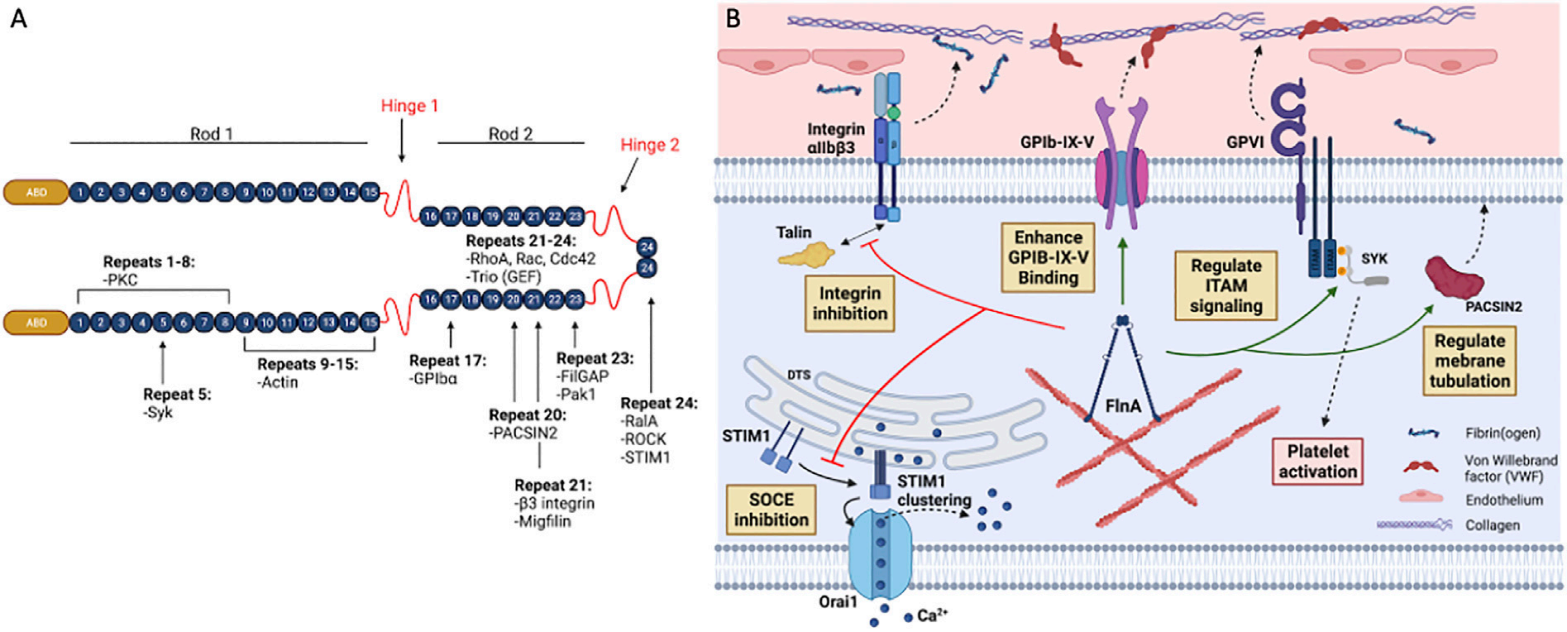
Structure and binding partners of FLNA. **(A)** FLNA is a 280 kDa homodimer consisting of an actin-binding domain (ABD) at the N-terminus, followed by 24 immunoglobulin (Ig)-like repeat domains folded into β-sheets (numbered 1–24). Two hinge domains, one at Ig repeat 15–16 and another at 23–24, separate the Ig domains into two different rod regions: rod one and rod 2. Rod one consists of Ig repeats 1–15, and rod two consists of Ig repeats 16–23. Dimerization occurs through the interaction of repeat 24. FLNA interacts with many receptors and signaling molecules through its 24 Ig repeats. **(B)** In platelets, the store-operated calcium entry (SOCE) is regulated by the interaction between STIM1 and Orai1. Upon depletion of Ca^2+^ storage, STIM1 undergoes a conformational change and multimerizes on the dense tubular system (DTS) membrane. Consequently, STIM1 clusters initiate the assembly of Orai1 subunits in the plasma membrane, forming a Ca^2+^ channel which leads to the influx of extracellular Ca^2+^. FLNA downregulates SOCE function by directly interacting with STIM1 in the actin cytoskeleton, thereby abolishing the STIM1-Orai1 interaction. In resting platelets, FLNA has been proposed to constitutively associate with integrin β3 cytoplasmic tail (CT) through its Ig repeat 21. This association blocks the interaction between the β3 CT and talin or kindlin-3, thereby inhibiting integrin αIIbβ3 activation. GPIb-IX-V mediates adhesion of platelets to von Willebrand factor (VWF) upon endothelial injuries. FLNA constitutively interacts with GPIb-IX-V and enhances its binding to VWF. This interaction involves FLNA Ig repeat 17 to the GPIbα CT of GPIb-IX-V. FLNA also positively regulates ITAM- and ITAM-like-containing receptor signaling in platelets by interacting with Spleen tyrosine kinase (Syk). This interaction is essential for GPVI receptor signaling, which is an important pathway for collagen-mediated platelet adhesion and activation. FLNA Ig repeat 20 interacts with the F-BAR protein PACSIN2 to regulate membrane tubulation and intracellular membrane architecture in platelets. This interaction also likely contributes to demarcation membrane system (DMS) formation in megakaryocytes. Figure created with BioRender.com.

FLNA is a 280-kDa protein comprised of an N-terminal actin-binding domain followed by 24 immmunoglobulin (Ig)-like repeats of beta (β)-sheets containing seven β-strands (A-G) each **(** Figure 1A). The N-terminal spectrin-related actin-binding domain contains two calponin homology domains (CH1 and CH2), and another distal secondary actin binding domain (Robertson et al., 2003; Nakamura et al., 2007). The FLNA molecule is organized into rod one containing repeats 1–15; rod two containing repeats 16–23; and two calpain-sensitive flexible loops, called hinges, separating rods one and two and repeats 23 and 24, respectively (Figure 1A). While rod one is a 58 nm extended chain, interactions between repeats 16, 18, 20 with repeats 17, 19, and 21, respectively, lead to the compact propeller-like structure of rod 2 (Nakamura et al., 2007; Ruskamo et al., 2012; Tossavainen et al., 2012). FLNA forms a V-shape upon self-dimerization at the C-terminal repeat 24 (Figure 1A), and the two actin-binding domains bind and organize actin into an orthogonal network. Rod two mediates binding to multiple proteins (Table 1) thus contributing to the major scaffolding function of FLNA.

### Clinical implications of FLNA gene variants

Variants of the *FLNA* gene confer a group of clinical disorders collectively termed filaminopathies A (Nurden et al., 2011). The most prominent such disorder is X-linked periventricular heterotopia (PVNH), caused by a defect in neuronal migration during fetal development (Feng and Walsh, 2004; Robertson, 2005). PVNH manifests as cardiac and cerebral malformations and seizure disorders (Feng and Walsh, 2004). Frameshift, missense and nonsense mutations of *FLNA* underlie PVNH (Robertson, 2005). Other filaminopathies A include otopalatodigital syndrome, frontometaphyseal dysplasia and Melnick-Needles syndrome, which are characterized by skeletal dysplasia (Robertson, 2007). Multiple variants of the human *FLNA* gene are specifically associated with aberrant platelet function (Vassallo et al., 2020; Tanner et al., 2022). Consequently, patients with PVNH can exhibit hemorrhage, coagulopathy and thrombocytopenia (Nurden et al., 2011; Berrou et al., 2017).

### FLNA is a critical determinant of platelet structural integrity and shape change

The most compelling direct evidence of the critical role of FLNA in platelet function has been obtained from studies with FLNA knockout mice. Because FLNA deficiency is an embryonic lethal trait, conditional knockouts were generated where FLNA expression is specifically deleted in megakaryocytes and platelets (Falet et al., 2010; Jurak Begonja et al., 2011). These conditional knockout mice display macrothrombocytopenia, which is characterized by morphologically large platelets that circulate in low numbers (Falet et al., 2010; Jurak Begonja et al., 2011). Electron micrographs of resting platelets showed that actin filaments are dissociated from the cell membranes in FLNA-deficient platelets (Falet et al., 2010), indicating that FLNA is essential for maintaining structural continuity between the plasma membrane and the actin cytoskeleton. Importantly, FLNA-null platelets fail to assemble actin normally in response to stimulation by either thrombin or collagen-related peptide (Falet et al., 2010). In a separate study, the stability of the platelet plasma membrane was monitored (Jurak Begonja et al., 2011). After 24 h of storage, FLNA-deficient platelets exhibited increased microvesiculation relative to control platelets (Jurak Begonja et al., 2011), which further reinforces the notion that FLNA underpins membrane stability. Collectively, the available evidence clearly implicates FLNA as an essential transducer of PAR- and GPVI-driven signals to the actin cytoskeleton. Less clearly defined are the exact protein/protein interactions between FLNA and the various plasma membrane and cytoplasmic proteins that regulate the actin cytoskeleton.

## FLNA interactions with platelet plasma membrane proteins

Multiple receptors mediate the platelet response to vascular injury (Jurk and Kehrel, 2005); moreover, platelet function is directly contingent on the integrity of the actin cytoskeleton (Bearer et al., 2002; Sorrentino et al., 2015; Bender and Palankar, 2021). The receptors that drive platelet response to injury include the PARs which recognize thrombin (Coughlin, 2000), the glycoprotein Ib/V/IX complex which recognizes von Willebrand factor (vWF) (Shen et al., 2000; Zhang et al., 2022), and the glycoprotein VI (GPVI) receptor which recognizes exposed collagen following vascular injury (Jurk and Kehrel, 2005). FLNA is essential for normal PAR4-and GPVI-driven signal transduction (Falet et al., 2010) and also directly binds other receptors thus serving as a critical signaling conduit between the plasma membrane and the platelet cytoskeleton (Figure 1B).

### FLNA interaction with GPIbα

The initial platelet adhesion to the damaged vascular wall requires the binding of von Willebrand factor (vWF) to the GPIb-IX-V receptor complex at the platelet surface (Varga-Szabo et al., 2008a). This quadripartite complex consists of GPIbα, GPIbβ, GPIX, and GPV (Zhang et al., 2022); notably, FLNA constitutively binds GPIbα, thus anchoring the receptor complex to the actin cytoskeleton (Nakamura et al., 2006). Specifically, the hydrophobic region of FLNA Ig repeat 17 binds the cytoplasmic tail of GPIbα at amino acids 563–571 (Meyer et al., 1997; Cranmer et al., 2005; Nakamura et al., 2006). Multiple evidences support the functional importance of the FLNA-GPIbα interaction. For example, FLNA-deficient platelets exhibit abnormal surface expression and distribution of GPIbα (Falet et al., 2010). Moreover, transgenic mice expressing a mutant, non-FLNA-binding GPIbα produce platelets that fragment under shear stress (Cranmer et al., 2011). Similarly, Chinese hamster ovary (CHO) cells transfected with a non-FLNA-binding GPIbα failed to generate contractile forces on VWF substrates (Feghhi et al., 2016). Conversely, a gain-of-function mutation in FLNA Ig repeat 16 was proposed to promote the GPIbα-FLNA interaction and platelet adhesion on a VWF surface (Berrou et al., 2013). Combined with data suggesting that FLNA is required for normal GPIbα trafficking to the cell surface (Kanaji et al., 2012), it is therefore clear that the GPIbα-FLNA association is of crucial importance in the context of platelet adhesion and hemostasis. A recently published report implicates the GPIbα-FLNA interaction as a determinant in GPIbα receptor shedding, which diminishes the function of stored platelets (Zhou et al., 2022). The authors report that GPIbα receptor shedding is directly related to the stability of the actin cytoskeleton and the integrity of GPIbα-FLNA binding (Zhou et al., 2022), thus further underscoring the role of FLNA in platelet signal transduction.

### FLNA interaction with integrin αIIbβ3

Integrins are heterodimeric, transmembrane receptors that adopt a folded, closed conformation in the resting platelet. Integrins can be activated *via* their cytoplasmic domains (termed “inside-out signaling”) (Huang et al., 2019) or by ligand binding to their extracellular domain (“outside-in” signaling) (Shattil and Newman, 2004). FLNA interacts with integrin beta (β) subunits. This interaction requires FLNA Ig repeat 21 and a region located between two endocytic NPxY/F motifs on β subunits that also interact with talin-1 (Kiema et al., 2006) and kindlin-3 (Yates et al., 2012).

Megakaryocytes and platelets express both β1 and β3 integrins: the collagen receptor α2β1, the fibronectin receptor α5β1, the laminin receptor α6β1, the fibrinogen receptor αIIbβ3, and the vitronectin receptor αVβ3 [recently reviewed in (Yang et al., 2022)]. While the β3 subunit does not have an optimal FLNA-binding motif (unlike the β1 subunit), most studies on platelets have focused on the interaction between FLNA and αIIbβ3 and its role in fibrinogen binding and platelet aggregation. As the fibrinogen-binding receptor (Bennett et al., 2009; Nieswandt et al., 2009; Jackson and Schoenwaelder, 2010), the glycoprotein IIb/IIIa (αIIbβ3 integrin) receptor is critical for normal platelet function and transmits signals to/from the platelet actin cytoskeleton (Morse et al., 2014). Two cytoplasmic proteins, talin (Petrich et al., 2007a; Petrich et al., 2007b) and kindlin-3 (Moser et al., 2008) are identified as integrin “activators” that bind the cytoplasmic domain of the β3 integrin subunit, triggering conformational changes that expose the integrin’s extracellular ligand binding site (Ginsberg, 2014) to promote integrin activation (Ma et al., 2007). Notably, the Ig repeat 21 of FLNA also binds the β3 integrin at amino acids 747–755, thus competing with talin and kindlin-3 (Kiema et al., 2006; Liu et al., 2015; Rosa et al., 2019).

The prevailing theory regarding integrin activation is that FLNA binding to the β3 integrin serves primarily to retain the latter in a resting state (Liu et al., 2015), and that the dissociation of FLNA from the integrin promotes talin and kindlin binding to, and activation of, the integrin (Ithychanda et al., 2009). This contention is partially supported by a report of increased β3 integrin function conferred by a *FLNA* variant near the C-terminus (repeat 24) carried by a human subject (Berrou et al., 2017). The patient’s platelets exhibited increased aggregation, secretion, and αIIbβ3 integrin activity, as well as an increased association between talin and the β3 subunit (Berrou et al., 2017). The same research group recently generated a knock-in mouse that recapitulates the FLNA mutation; platelets from this knock-in mouse essentially replicate the gain-of-function phenotype observed in the human subject (Adam et al., 2022). These data clearly point to the functional importance of the FLNA-αIIbβ3 integrin association with regards to hemostasis and thrombosis. However, it should be noted that platelets completely devoid of FLNA exhibit comparable, yet not elevated, αIIbβ3 integrin activity relative to controls (Falet et al., 2010). This finding suggests that the regulation of integrin activity is perhaps more complex than is currently known, and that further research is required to fully validate the hypothesized role of FLNA as a strict suppressor of integrin activation.

### FLNA interaction with PACSIN2

FLNA interacts with the adaptor protein PACSIN2, a member of the Bin/amphiphysin/Rvs (BAR) family of proteins that bind and tubulate membranes (Begonja et al., 2015). PACSIN2 has been implicated in receptor internalization, caveolae biogenesis, endosomal trafficking, and cell adhesion, spreading, and migration. In platelets, PACSIN2 colocalizes with GPIbα in membrane invaginations reminiscent of the open canalicular system (OCS), the membrane reservoir for platelet spreading and channels for granule secretion following platelet activation (Heijnen and van der Sluijs, 2015). In megakaryocytes, PACSIN2 colocalizes with the initiating demarcation membrane system (DMS), the highly invaginated membrane system that provides membrane for future platelets. This interaction requires FLNA Ig repeat 20 and the tip of PACSIN2 F-BAR domain, and regulates membrane tubulation *in vitro*, in platelets, and in megakaryocytes (Begonja et al., 2015). Single nucleotide polymorphisms in *PACSIN2* have been associated with platelet count and size (Astle et al., 2016; Eicher et al., 2016; Chen et al., 2020; Vuckovic et al., 2020).

### FLNA interactions with G-protein coupled receptors (GPCRs)

In addition to directly binding GPIbα and integrin αIIbβ3, FLNA also transduces signals from other platelet receptors. For example, thrombin is a serine protease and a potent physiological agonist that activates platelets at concentrations as low as 0.1 nM (Greco et al., 1995; Michelson, 2013). Thrombin signals *via* PARs (Vu et al., 1991), which are seven-transmembrane G-protein coupled receptors (GPCRs) (Michelson, 2013). Other platelet GPCRs include the P2Y_12_ receptor, which recognizes ADP, and the thromboxane receptor (TP receptor), which recognizes thromboxane A2 (Jurk and Kehrel, 2005; Dowal and Flaumenhaft, 2010). As mentioned in Section 1 above, PAR-driven signaling is thought to elicit changes in the actin cytoskeleton *via* G_q_, PLCβ, rise in [Ca^2+^]_i_ and PKC activation. However, a model has been proposed in which Ig repeat 21 of FLNA binds GPCRs with high affinity (Tirupula et al., 2015). Although this model has not been specifically validated in platelets, it does raise the interesting possibility that FLNA modulates thrombin-induced shape change *via* direct bridging of PARs with the actin cytoskeleton.

## FLNA regulation of the platelet cytoskeleton *via* cytosolic proteins

### FLNA regulation of Ca^2+^ signaling

The rise in intracellular calcium ([Ca^2+^]_i_) following platelet activation is essential for actin assembly. This is exemplified by data indicating that platelets fail to spread in the presence of the Ca^2+^-chelating agent BAPTA/AM (Mazharian et al., 2007). Accordingly, the [Ca^2+^]_i_ rise is necessary for complete platelet aggregation and thrombus formation (Jardin et al., 2007; Varga-Szabo et al., 2008b). Interestingly, FLNA was recently found to regulate Ca^2+^ signaling in platelets. Lopez et al. reported that stromal interaction molecule 1 (STIM1), which serves as a calcium-sensing molecule at the endoplasmic reticulum (ER) (Lunz et al., 2019), co-immunoprecipitates with FLNA in thapsigargin-treated human platelets. Following activation-induced Ca^2+^ release from intracellular stores, ER-localized STIM1 interacts with Orai1, a Ca^2+^ release-activated channel on the plasma membrane (Galan et al., 2011). This interaction promotes store-operated calcium entry (SOCE) and the Ca^2+^-dependent platelet functions. Further, the authors reported that following siRNA knockdown of FLNA in platelets, the STIM1-Orai1 interaction (and the Ca^2+^) rise was accentuated. The authors conclude that FLNA regulates store-operated calcium entry (SOCE) process by restraining rises in [Ca^2+^]_i_ thus avoiding Ca^2+^ overloading (Lopez et al., 2018). The finding of increased [Ca^2+^]_i_ in the FLNA-knockdown platelets appears to stand in contrast with data obtained from FLNA-deficient mouse platelets, in which actin assembly is diminished (Falet et al., 2010). It is possible that FLNA’s modulation of [Ca^2+^]_i_ fluxes is independent of its role in modulating shape change. Additional research is required to fully dissect the role(s) of FLNA in this complex biological system.

### FLNA interactions with protein kinases

As noted in Table 1, FLNA interacts with multiple signaling molecules that modulate actin assembly. Syk is a tyrosine kinase that is a critical element of the GPVI-driven signaling pathway (Manne et al., 2015). Stimulation of ITAM- and hemITAM–containing receptors GPVI or CLEC2 lead to their phosphorylation and recruitment and activation of the tyrosine kinase Syk (Poole et al., 1997; Suzuki-Inoue et al., 2006). Syk then promotes activation of downstream signaling leading to increases in [Ca^2+^]_i_ and inside-out activation of αIIbβ3 integrin (Ozaki et al., 2005). Notably, Syk was shown to be essential for lamellipodial formation and platelet spreading (Hughan et al., 2007). Moreover, FLNA Ig repeat 5 binds directly to Syk and regulates the ITAM-Syk signaling pathway by promoting Syk recruitment to the plasma membrane (Falet et al., 2010). Another kinase, protein kinase C (PKC), is activated by rises in [Ca^2+^]_i_ and has been shown to regulate actin assembly in platelets (Harper and Poole, 2007; Harper and Poole, 2010). Interactions between FLNA and PKC have been documented in fibroblasts (Glogauer et al., 1998) and in HeLa cells (Tigges et al., 2003), and evidence also indicates that FLNA and PKC share a common function in regulating cell spreading (Kim et al., 2010) although this was not directly tested in platelets. Nevertheless, these data collectively support the notion that FLNA likely integrates multiple intracellular kinase pathways that regulate the actin cytoskeleton.

### Other FLNA interacting proteins not documented in platelets

In addition to the proteins described above, there are multiple FLNA interacting proteins whose interactions with FLNA have not been specifically documented in platelets to date. Of particular relevance to actin cytoskeletal dynamics are the Rho GTPases. The formation of the cell membrane extensions characteristic of activated platelets (e.g. filopodia and lamellipodia) is catalyzed by the Rho GTPases Cdc42 and Rac1 (Nobes and Hall, 1995). Since Cdc42 and Rac1 reportedly bind FLNA Ig repeat 24 (Ohta et al., 1999), it is reasonable to speculate that FLNA directly modulates their activity since actin assembly is curtailed in FLNA-deficient platelets relative to controls (Falet et al., 2010). Conversely, another FLNA binding partner is FilGAP, which inactivates Rac1 (Ohta et al., 2006). FLNA binds FilGAP at the Ig repeat 23 and regulates FilGAP activity in cells (Ohta et al., 2006), possibly by approximating FilGAP and Rac1. These data suggest that FLNA may serve to constrain actin assembly by facilitating the inactivation of Rac1. Further work is clearly required to elucidate the precise determinants through which FLNA regulates actin dynamics in platelets.

BOX 2 **Key points: FLNA as a plasma membrane-cytoskeleton scaffold**

• Normal actin assembly in platelets is disrupted in the absence of FLNA.
• FLNA tethers platelet GPIbα, integrin αIIbβ3 (and possibly GPCRs) to the actin cytoskeleton.
• There exist a multitude of FLNA interacting proteins that regulate the cytoskeleton (although not all interactions with FLNA have been demonstrated in platelets).

## Perspectives

Much is now known regarding the critical importance of FLNA in maintaining the integrity of both the plasma membrane and the cytoskeleton, and for relaying critical signals between these two structures. Data from platelets further underscore how FLNA integrates signaling pathways between the plasma membrane and the actin cytoskeleton, and also provide an explanation for the coagulopathies associated with *FLNA* gene variants.

Despite the advances in knowledge, open questions remain. For example, FLNA seemingly functions to suppress integrin αIIbβ3 function yet this integrin is not overactive in FLNA-null mouse platelets. Moreover, it is clear that FLNA is required for spreading of activated platelets although in some experiments, FLNA appears to constrain the [Ca^2+^]_i_ rise that is normally required for actin assembly. Therefore, considerable additional research is still required to fully unravel the many facets of FLNA function in platelets and other cell types. This is particularly relevant given the large (and growing) number of proteins that interact with FLNA. Ultimately, an improved understanding of the FLNA-centric signaling networks, combined with detailed structural information on the specific protein-protein interactions, should identify viable therapeutic targets for managing coagulopathies and other diseases.
